# Feasibility of a Remote Patient Video Education Intervention to Improve Care Coordination for Rural Cancer Patients

**DOI:** 10.1007/s13187-024-02550-6

**Published:** 2024-12-05

**Authors:** Izumi Okado, Christa Braun-Inglis, Kehau Matsumoto, Carry Elhajj, Kevin Cassel, Jeffrey Berenberg, Randall F. Holcombe

**Affiliations:** 1 University of Hawai’i Cancer Center, 701 Ilalo St., Honolulu, HI 96813 USA; 2 University of Vermont Cancer Center, Burlington, VT USA

**Keywords:** Cancer, Healthcare delivery, Care coordination, Rural cancer care delivery

## Abstract

Cancer patients residing in rural areas experience substantial barriers to care and suboptimal care coordination. To date, there is a paucity of interventions to improve care coordination for rural cancer patients. In this study, we conducted a pilot trial to assess the feasibility and efficacy of a remote, tablet-based patient video education intervention focused on cancer care coordination among rural patients in Hawaii. The pilot trial utilized a single-arm, pre-post intervention design. Our TED-talk style video education intervention included talks on cancer basics, care coordination, and self-advocacy. Eligible participants were rural patients newly diagnosed with early-stage cancer receiving adjuvant therapy. Validated instruments were administered at baseline and post-intervention to assess patients’ perceptions of care coordination and self-advocacy. Acceptability and satisfaction were assessed using semi-structured interviews. Descriptive statistics were used to describe study outcomes. From January 2022 to December 2022, 19 patients enrolled on the study; the mean age was 52.2. Participants were racially diverse. A total of 71.4% of eligible patients completed all assessments. No changes were observed in the overall perceptions of care coordination. However, improved scores were observed for the care coordination instrument communication domain (Cohen’s *d* =  − 0.76, 95% CI: − 1.45, − 0.03). There was a trend for improved scores on navigation and self-advocacy. All participants reported high satisfaction with the intervention. Results support the feasibility, satisfaction, and acceptability of this intervention among rural cancer patients in Hawaii. Further study is needed to evaluate the intervention in other rural areas. **Clinical Trial Registration:** NCT05162404.

**Registration Date:** 12/17/2021.

## Introduction

Rural residents have disproportionately higher all cancer mortality rates than those residing in urban areas [1]. Higher cancer mortality rates among rural residents are thought to be due, in part, to care delivery challenges in rural areas. Effective care coordination is a key component of high-quality, patient-centered cancer care [2]; however, prior research indicates that rural cancer patients experience suboptimal care coordination [3–6]. Despite increased efforts to address rural disparities in cancer care delivery, there is a paucity of interventions to improve care coordination for rural cancer patients.

Rural cancer patients face substantial barriers to cancer care. Compared to urban/nonrural areas, rural health care systems are more geographically dispersed, and there are fewer oncology providers and limited treatment facilities and resources in rural communities [7]. As a result, many rural residents must travel over 50 miles to obtain cancer-related services, incurring additional travel-related financial burdens [8, 9]. Moreover, rural residents are at higher risk for social disadvantages including lack of insurance/underinsurance, poverty, lower educational attainment, and low or moderate health literacy [7, 8, 10]. Notably, low health literacy has been linked to differential access to health care, patient reports of poor care coordination, and low satisfaction with care [6, 11–13]. Rural cancer patients are more likely to experience health literacy-related barriers, and a lack of patients’ understanding of cancer and care processes may further exacerbate care coordination-related challenges for rural patients [5, 6].

One approach to improve care coordination experiences for rural cancer patients is to increase patients’ knowledge about cancer and care coordination. Many cancer education materials have been previously developed, and systematic reviews have shown that patient education, particularly video-based interventions, improves knowledge and satisfaction and reduces psychological distress [14, 15]. Cancer education materials are also widely available on the internet. However, most prior interventions have been developed and tested in urban/academic cancer centers, and for rural patients, lack of resources (e.g., internet access, computer) may hinder access to internet-based information. Further, a meta-analysis of internet use among cancer patients has found that some patients who sought cancer-related information online have found the information as overwhelming and/or confusing [16]. Our preliminary research has also found that while lack of knowledge adversely impacted rural cancer patients’ perceptions of care coordination, the ability to self-advocate for one’s care had a positive impact on care coordination [5]. Enhancing self-advocacy may be particularly salient for rural patients, as the ability to self-advocate may increase active engagement in treatment decision-making and more effective communication with members of one’s healthcare team [17].

A patient-centered education intervention designed specifically for rural cancer patients has the potential to improve patients’ perceptions of care coordination and enhance self-advocacy. In this study, we assessed the feasibility and efficacy of a remote, tablet-based patient video education intervention focused on cancer care coordination among rural patients in Hawaii.

## Methods

### Study Design

This study utilized a single-arm, pre-post intervention study design to pilot test a novel, TED-talk style patient video education intervention. The trial was conducted remotely by the research team at the University of Hawaii Cancer Center (UHCC). Recruitment occurred from January 2022 to December 2022; all follow-up assessments were completed in March 2023. Participants were referred from rural oncology providers or navigation teams. Following consent, participants completed the baseline assessment and received an Amazon Fire tablet pre-loaded with the intervention materials by mail or in-person where a navigation team staff was available at rural oncology clinics. Participants completed post-intervention measures and follow-up telephone interviews with the study team 4–6 months following the start of adjuvant therapy. The baseline and post-intervention measures were available as paper-based surveys. Study procedures were reviewed and approved by the UHCC Protocol Review and Monitoring Committee and the WCG IRB. All participants provided written informed consent. The trial information is available on ClinicalTrials.gov (Identifier: NCT05162404).

### Participants

Patients residing in rural areas of Hawaii were eligible to participate in the study. Rural areas were defined using the Rural–Urban Commuting Area Codes (RUCA; rural ≥ 4) and included islands of Kauai, Maui, and the Hawaii Island. Additional eligibility criteria were a new diagnosis of early-stage cancer (I-III), ability to read/speak English, and planned adjuvant therapy or treatment initiation within the preceding 1 month. Patients were excluded if they had a prior history of malignancy within the past 3 years.

### Intervention

Following preliminary research and literature review, we developed a TED-talk style patient video education program. In our preliminary research, lack of information about cancer and treatment options and the need to self-advocate for care were identified as potential targets of intervention to improve patients’ perceptions of care coordination [5]. The intervention entailed viewing of the three main videos developed for this study: (1) cancer basics, (2) care coordination, and (3) self-advocacy. Each module was 15–20 min and presented in a TED-talk style by oncology clinicians (a medical oncologist and an advanced practice provider) and a patient advocate. A TED-talk style was selected as the platform, as it is a popular presentation style where a presenter delivers an engaging talk on a specific topic with accompanying slides/images projected on a large screen in front of live audience members. Content of care coordination and self-advocacy modules were tailored to address specific concerns and issues that rural-residing patients may encounter while seeking cancer treatment. All videos were pre-loaded on an Amazon Fire tablet, so internet access was not required to participate in the study.

Videorecording was conducted in a large conference room at the University of Hawaii Cancer Center, with interested patients, survivors, and family members attending the talks in-person. Additionally, following each talk, Q&A sessions were conducted, where audience members posed questions/comments to each speaker. Q&A sessions were also available on the tablet as optional video contents.

### Measures

#### Primary Outcome

The primary outcome of this study was feasibility, as defined as the proportion of patients who enrolled on the study and completed both baseline and post-intervention assessments. Additionally, patients’ perceptions of feasibility were assessed during the post-intervention telephone interview.

#### Secondary Outcomes

The secondary outcome measures evaluated the effect of the intervention on patients’ perceptions of care coordination and self-advocacy. Patients’ perceptions of care coordination were assessed by the Care Coordination Instrument (CCI). This 29-item instrument assesses overall perceptions of care coordination and across three domains: communication, navigation, and operational [18]. The CCI has been used in prior care coordination studies and has demonstrated excellent psychometric properties [18–20]. Items are scored on a 4-point Likert-style scale (strongly disagree to strongly agree), with higher scores indicating positive perceptions of care coordination. Self-advocacy was measured using the adapted Cancer Self-Advocacy Scale. The Female Self-Advocacy in Cancer Survivorship Scale (FSACS) was adapted and modified for use in rural cancer patients [21]. This 20-item instrument measures self-advocacy and includes the overall scores and three subscales: informed decision making, effecting communication, and connected strength. Modifications to the FSACS instructions and select items included adjusting specific references regarding “female cancer survivors” to “cancer patients,” improving readability of the items (e.g., shortening longer questions, using clearer phrasing), and including reverse-coded items. Adaptations were made by the study team in consultation with the researcher who developed and validated the FSACS.

Additional outcome measure included patients’ satisfaction with the intervention, as assessed by semi-structured telephone interviews post-intervention.

### Statistical Analysis

The endpoints were assessed at 4–6 months following the start of adjuvant therapy. Descriptive statistics were used to describe differences in care coordination and self-advocacy scores between baseline and post-intervention. Qualitative responses to the post-intervention telephone interviews were transcribed and summarized.

## Results

Between January 2022 and December 2022, 19 patients consented to participate in the study (21 approached); 71.4% (*n* = 10) of eligible patients completed all components of the study. Five patients (26%) became ineligible after enrolling on the study (disease progression, relocation) and were withdrawn. No major differences in demographic characteristics were observed between participants who completed the intervention and those who were withdrawn.

A total of 74% of participants were female, and the mean age was 52.2 (*SD* = 15.8). A total of 42.1% self-identified as two or more races, 21.1% as White, 15.8% as Japanese, and 10.5% as Filipino. The most common cancer types were breast and lung (47.4% and 15.8%, respectively). Regarding education status, 31.6% reported completing some college, 26.3% high school/GED, and 26.3% graduate school.

The descriptive statistics for the two secondary outcome measures, perceptions of care coordination and self-advocacy, are shown in Table 1. Only those who completed all assessments were included in the analysis (*n* = 10). There were no overall changes in the perceptions of care coordination (CCI total scores) between baseline and post-intervention. Improved scores were observed for the CCI communication domain (+ 4.5; Cohen’s *d* =  − 0.76, 95% CI: − 1.45, − 0.03). Post-intervention, there was a trend for improved scores for the navigation domain, whereas a trend for lower scores was observed for the operational domain. For self-advocacy, there was an overall trend for improved scores for the total and informed decision making and connected strengths subscales. Effecting communication subscale scores remained unchanged at post-intervention.

**Table 1:** Secondary outcome measures and descriptive statistics

Measure (*n* = 10)	Mean	Cohen’s *d*	95% CI
CCI total	Mean score at baseline 58.7	0	− 0.62, 0.62
	Change at post-intervention 0		
Communication	Mean score at baseline 29.3	− 0.76	− 1.45, − 0.03
	Change at post-intervention + 4.5		
Navigation	Mean score at baseline 24.4	− 0.42	− 1.06, 0.24
	Change at post-intervention + 1.6		
Operational	Mean score at baseline 21.9	0.31	− 0.34, 0.93
	Change at post-intervention − 1.2		
CSAS total	Mean score at baseline 92.3	− 0.26	− 0.89, 0.38
	Change at post-intervention + 2.6		
Informed decision making	Mean score at baseline 31.2	− 0.25	− 0.88, 0.38
	Change at post-intervention + 1.4		
Effecting communication	Mean score at baseline 28.5	− 0.12	− 0.74, 0.51
	Change at post-intervention + 0.4		
Connected strengths	Mean score at baseline 32.6	− 0.28	− 0.90, 0.36
	Change at post-intervention + 0.8		

Note: *CCI*, care coordination instrument; *CSAS*, Cancer Self-Advocacy Scale

Post-intervention, semi-structured telephone interviews were conducted by the study team to assess patients’ perceptions of feasibility, satisfaction, and acceptability of the intervention. Among participants, 100% reported that they were satisfied with the intervention, with 60% indicating high satisfaction (the highest score on a 4-point Likert scale). All participants reported finding the intervention as useful, with 80% indicating “very useful.” A total of 90% indicated that navigating the tablet was easy or very easy. A total of 70% indicated that the video content was applicable to their care. All participants indicated that they would recommend the videos to other cancer patients. In response to open-ended questions probing for feedback on the intervention, participants described the videos as easy to understand and informative, and many found the self-advocacy module to be most helpful. Additionally, multiple participants indicated that they had a positive experience with participating in the study, and being able to view the videos more than once was a benefit of a video-based education. Exemplar quotes are shown in Table 2.

**Table 2:** Exemplar quotes from post-intervention telephone interviews

Questions	*Exemplar quotes*
Which aspects of the videos were most useful?	“Easy to understand something so complex as cancer”
	“All the videos were informative…I liked that I could go back and watch again.”
	“Most useful part…one lady talked a bit from her own experience with care, talk to others.”
	“Encouraging the patient to be motivated to ask questions and seek advice from their physicians.”
Additional comments	“Very informative. Enjoyed participating. Very helpful, videos talked about what’s needed.”
	“I enjoyed participating. Caring, very easy and comfortable.”
	“I feel that the videos should be shared with cancer patients as soon as they know that they have cancer or suspect they have cancer.”
	“Understanding what is cancer video should be available to everyone”

## Discussion

To address the need to improve rural cancer care delivery, we developed and pilot-tested a TED-talk style patient video education program designed specifically for rural patients with cancer. Findings suggest the feasibility and utility of this educational intervention for rural cancer patients. Although the overall perceptions of care coordination remained unchanged, we found an improved perception of communication and a trend for improved self-advocacy. Overall, participants reported high satisfaction with the program and the ease of a tablet-based education program.

In this study sample, 71% of eligible patients completed the education program and all assessments, indicating the feasibility of a remote, tablet-based education intervention for rural cancer patients. Follow-up interviews with the participants demonstrated that the program was feasible, and the use of a tablet device did not pose any difficulty. Although the majority of the participants completed the study, five patients (26%) were withdrawn after initial enrollment due to no longer meeting eligibility. Reasons for withdrawal included relocation, disease progression, and delays in treatment. These may be inherent challenges in conducting a clinical research study in rural settings, particularly where there is no clinical trial infrastructure. However, these barriers may suggest the potential advantage of a remote, tablet-based intervention to provide evidence-based cancer-related information to rural patients. A mobile, tablet-based platform allows for greater flexibility in how education intervention materials are provided to residents in rural and/or remote areas.

Notably, the results indicate improved perceptions regarding communication aspects of care coordination. Improved perceptions of communication may be in part due to increased knowledge about cancer and care coordination, mirroring prior findings in the literature on the link between health literacy and care coordination [6, 11]. Further, there were trends toward improved navigation and self-advocacy. It is possible that the self-advocacy talk emphasizing pro-active communication and discussing questions with oncology providers/team had facilitated better care coordination experiences for the participants. Some participants expressed feeling empowered by viewing the presentation by the patient advocate, and this may have led to increased engagement with their care and treatment decision-making.

Overall, we found high patient satisfaction with the program. Based on the post-intervention telephone interviews, all participants indicated satisfaction with the program and found the videos as easy to understand and helpful. Further, some participants identified additional benefits of the video intervention format, including the ease of using a tablet and the ability to repeat viewing of the videos. Moreover, the self-advocacy talk presented by a patient advocate was identified by some participants as being the most helpful.

Despite the strong positive feedback from the participants, it is important to note that there were substantial challenges in conducting a pilot trial in rural areas of Hawaii. At the time of this study, there was no clinical trial infrastructure established in these areas. None of the local clinics were clinical trial sites; thus, clinical research staff was not available at local clinics. In addition, our recruitment efforts were hampered by care delivery challenges. For example, some patients experienced significant delays in starting their cancer treatment, and these delays resulted in some participants being withdrawn from the study. These challenges underscore the need to increase access to cancer care and clinical trial infrastructure in rural areas.

There are some additional limitations of this study. First, due to the study design and a small sample size, we were unable to conduct hypothesis testing. Although the study sample was small, rural patients in Hawaii experience many similar challenges in care coordination with other rural patients in the USA including long travel distance to care and limited specialty care in local clinics. Further research with a larger sample and in other rural locations is needed to determine the efficacy of the intervention. Second, over half of the study participants had reported attaining higher education (college or graduate school), and this may not be representative of rural populations generally. Third, patients’ income was not collected as part of this study. As prior research has shown that income is a predictor of clinical trial participation [22], further research incorporating income as a covariate is needed.

## Conclusions

Among cancer patients in rural areas of Hawaii, our findings suggest the feasibility, satisfaction, and acceptability of a tablet-based patient video education intervention. Although the overall perceptions of care coordination remained comparable following the intervention, our results suggest that the intervention may improve communication regarding care coordination and self-advocacy. Further study is needed to evaluate the efficacy of the intervention in other rural areas.

## References

[1] Henley SJ, Anderson RN, Thomas CC et al (2017) Invasive cancer incidence, 2004–2013, and deaths, 2006–2015, in nonmetropolitan and metropolitan counties—United States. MMWR Surveill Summ 66(14):1 10.15585/mmwr.ss6614a1 PMC5879727 28683054

[2] IOM (2013) Delivering high-quality cancer care: charting a new course for a system in crisis. In: Levit L, Balogh E, Nass S, et al (eds) Delivering High-Quality Cancer Care: Charting a New Course for a System in Crisis. Washington (DC). 10.1200/JCO-24-01243 PMC11654450 39356979

[3] Okado I, Liu M, Elhajj C et al (2024) Patient reports of cancer care coordination in rural Hawaii. J Rural Health 1–7. 10.1111/jrh.12821. 10.1111/jrh.12821 38225683

[4] Okado I, Liu M, Elhajj C et al (2024) Time to diagnosis and treatment initiation during the COVID-19 pandemic among rural patients with cancer. Asia Pacific J Public Health 36(4):387–390 10.1177/10105395241240968 38553966

[5] Chang S, Liu M, Braun-Inglis C et al (2024) Cancer care coordination in rural Hawaii: a focus group study. BMC Health Serv Res 24(1):518 38658990 10.1186/s12913-024-10916-1 PMC11043031

[6] Martinez-Donate AP, Halverson J, Simon NJ et al (2013) Identifying health literacy and health system navigation needs among rural cancer patients: findings from the Rural Oncology Literacy Enhancement Study (ROLES). J Cancer Educ 28(3):573–581 23813542 10.1007/s13187-013-0505-x PMC3755018

[7] Levit LA, Byatt L, Lyss AP et al (2020) Closing the rural cancer care gap: three institutional approaches. JCO Oncol Pract 16(7):422–430 32574128 10.1200/OP.20.00174

[8] Zahnd WE, Davis MM, Rotter JS et al (2019) Rural-urban differences in financial burden among cancer survivors: an analysis of a nationally representative survey. Support Care Cancer 27(12):4779–4786 30972645 10.1007/s00520-019-04742-z PMC6786922

[9] Baldwin L-M, Cai Y, Larson EH et al (2008) Access to cancer services for rural colorectal cancer patients. J Rural Health 24(4):390–399 19007394 10.1111/j.1748-0361.2008.00186.x PMC3103393

[10] Blake KD, Moss JL, Gaysynsky A et al (2017) Making the case for investment in rural cancer control: an analysis of rural cancer incidence, mortality, and funding trends. Cancer Epidemiol Biomarkers Prev 26(7):992–997 28600296 10.1158/1055-9965.EPI-17-0092 PMC5500425

[11] Hawley ST, Janz NK, Lillie SE et al (2010) Perceptions of care coordination in a population-based sample of diverse breast cancer patients. Patient Educ Couns 81(Suppl):S34-40 21074963 10.1016/j.pec.2010.08.009 PMC2997113

[12] Kim H, Xie B (2017) Health literacy in the eHealth era: a systematic review of the literature. Patient Educ Couns 100(6):1073–1082 28174067 10.1016/j.pec.2017.01.015

[13] Berkman ND, Sheridan SL, Donahue KE et al (2011) Low health literacy and health outcomes: an updated systematic review. Ann Intern Med 155(2):97–107 21768583 10.7326/0003-4819-155-2-201107190-00005

[14] Gysels M, Higginson IJ (2007) Interactive technologies and videotapes for patient education in cancer care: systematic review and meta-analysis of randomised trials. Support Care Cancer 15(1):7–20 17024500 10.1007/s00520-006-0112-z

[15] Champarnaud M, Villars H, Girard P et al (2020) Effectiveness of therapeutic patient education interventions for older adults with cancer: a systematic review. J Nutr Health Aging 24(7):772–78232744575 10.1007/s12603-020-1395-3

[16] Eysenbach G (2003) The impact of the Internet on cancer outcomes. CA Cancer J Clin 53(6):356–371 15224975 10.3322/canjclin.53.6.356

[17] Hagan TL, Donovan HS (2013) Self-advocacy and cancer: a concept analysis. J Adv Nurs 69(10):2348–2359 23347224 10.1111/jan.12084 PMC3640735

[18] Okado I, Cassel K, Pagano I et al (2020) Development and psychometric evaluation of a questionnaire to measure cancer patients’ perception of care coordination. BMC Health Serv Res 20(1):52 31964391 10.1186/s12913-020-4905-4 PMC6975072

[19] Okado I, Cassel K, Pagano I et al (2020) Assessing patients’ perceptions of cancer care coordination in a community-based setting. JCO Oncol Pract 16(8):e726–e733 32216713 10.1200/JOP.19.00509 PMC7427418

[20] Okado I, Pagano I, Cassel K, Holcombe RF (2020) Perceptions of care coordination in patient-family caregiver dyads. Support Care Cancer 29:2645–2652. 10.1007/s00520-020-05764-8 32970231 10.1007/s00520-020-05764-8

[21] Hagan TL, Cohen SM, Rosenzweig MQ et al (2018) The Female Self-Advocacy in Cancer Survivorship Scale: a validation study. J Adv Nurs 74(4):976–987 29117439 10.1111/jan.13498 PMC5844819

[22] Unger JM, Hershman DL, Albain KS et al (2013) Patient income level and cancer clinical trial participation. J Clin Oncol 31(5):536–542 23295802 10.1200/JCO.2012.45.4553 PMC3565180

